# Kambó as a drug that can induce psychotic or manic symptoms. A case report

**DOI:** 10.1192/j.eurpsy.2024.865

**Published:** 2024-08-27

**Authors:** E. Arroyo Sánchez, C. Díaz Mayoral, P. Setién Preciados

**Affiliations:** Hospital Universitario Príncipe de Asturias, Madrid, Spain

## Abstract

**Introduction:**

Kambó, also known as the “frog medicine,” is a traditional Amazonian medicine derived from the secretions of the Phyllomedusa bicolor tree frog. It has gained global attention for its purported therapeutic properties, including its use in addressing mental health issues. However, the psychiatric effects of kambó remain poorly understood, particularly concerning manic symptoms or psychosis.

**Objectives:**

The primary objective of this review is to comprehensively analyze and evaluate the available literature regarding the connection between kambó use and psychosis or manic symptoms. Specifically, this review seeks to determine the prevalence of psychosis among kambó users, identify potential risk factors for the development of psychosis or manic symptoms in this context, explore the mechanisms underlying any observed psychiatric effects, and provide insights into the clinical implications of kambó use.

**Methods:**

A case report of a 34-year-old man with chronic delusional disorder who presented to the emergency department with manic symptoms coinciding in time with the use of Kambó.

**Results:**

The findings of this bibliographical review suggest that there is limited empirical evidence to establish a direct link between kambó use and psychosis. Most available studies are anecdotal or based on qualitative reports, making it challenging to draw definitive conclusions. While some case reports and interviews suggest that kambó use may be associated with transient psychotic-like symptoms, including visual and auditory hallucinations, more rigorous research is needed to confirm and characterize these effects. Several case reports and qualitative studies suggest that individuals who have undergone kambó ceremonies may experience transient manic-like symptoms, such as elevated mood, increased energy, and impulsivity. However, these reports lack systematic assessment and standardized measurement of manic symptoms. Mechanisms underlying these effects remain speculative, with some researchers proposing altered neurotransmitter systems as a potential explanation.

**Conclusions:**

In conclusion, this review underscores the scarcity of scientific literature on the potential association between kambó use and psychosis or maniac symptoms. Although anecdotal reports and qualitative studies suggest a link, there is a notable lack of robust empirical research to support or refute this claim. Future research should focus on conducting controlled studies to elucidate the psychiatric effects of kambó, including its potential to induce psychosis and maniac symptoms, while also considering cultural and individual factors that may influence outcomes. Such research would contribute to a more comprehensive understanding of kambó’s psychopharmacological profile and its implications for mental health.

**Disclosure of Interest:**

None Declared

